# Non-Canonical Smads Phosphorylation Induced by the Glutamate Release Inhibitor, Riluzole, through GSK3 Activation in Melanoma

**DOI:** 10.1371/journal.pone.0047312

**Published:** 2012-10-12

**Authors:** Walid Abushahba, Oyenike O. Olabisi, Byeong-Seon Jeong, Rajeev K. Boregowda, Yu Wen, Fang Liu, James S. Goydos, Ahmed Lasfar, Karine A. Cohen-Solal

**Affiliations:** 1 Department of Medicine, Division of Medical Oncology, University of Medicine and Dentistry of New Jersey - Robert Wood Johnson Medical School, The Cancer Institute of New Jersey, New Brunswick, New Jersey, United States of America; 2 Department of Surgery, Division of Surgical Oncology, University of Medicine and Dentistry of New Jersey - Robert Wood Johnson Medical School, The Cancer Institute of New Jersey, New Brunswick, New Jersey, United States of America; 3 Center for Advanced Biotechnology and Medicine, Susan Lehman Cullman Laboratory for Cancer Research, Department of Chemical Biology, Ernest Mario School of Pharmacy, Rutgers, The State University of New Jersey, Piscataway, New Jersey, United States of America; 4 Department of Pharmacology and Toxicology, Ernest Mario School of Pharmacy, Rutgers, The State University of New Jersey, Piscataway, New Jersey, United States of America; University of Sherbrooke, Canada

## Abstract

Riluzole, an inhibitor of glutamate release, has shown the ability to inhibit melanoma cell xenograft growth. A phase 0 clinical trial of riluzole as a single agent in patients with melanoma resulted in involution of tumors associated with inhibition of both the mitogen-activated protein kinase (MAPK) and phophoinositide-3-kinase/AKT (PI3K/AKT) pathways in 34% of patients. In the present study, we demonstrate that riluzole inhibits AKT-mediated glycogen synthase kinase 3 (GSK3) phosphorylation in melanoma cell lines. Because we have demonstrated that GSK3 is involved in the phosphorylation of two downstream effectors of transforming growth factor beta (TGFβ), Smad2 and Smad3, at their linker domain, our aim was to determine whether riluzole could induce GSK3β-mediated linker phosphorylation of Smad2 and Smad3. We present evidence that riluzole increases Smad2 and Smad3 linker phosphorylation at the cluster of serines 245/250/255 and serine 204 respectively. Using GSK3 inhibitors and siRNA knock-down, we demonstrate that the mechanism of riluzole-induced Smad phosphorylation involved GSK3β. In addition, GSK3β could phosphorylate the same linker sites *in vitro*. The riluzole-induced Smad linker phosphorylation is mechanistically different from the Smad linker phosphorylation induced by TGFβ. We also demonstrate that riluzole-induced Smad linker phosphorylation is independent of the expression of the metabotropic glutamate receptor 1 (GRM1), which is one of the glutamate receptors whose involvement in human melanoma has been documented. We further show that riluzole upregulates the expression of INHBB and PLAU, two genes associated with the TGFβ signaling pathway. The non-canonical increase in Smad linker phosphorylation induced by riluzole could contribute to the modulation of the pro-oncogenic functions of Smads in late stage melanomas.

## Introduction

Transforming growth factor-beta (TGFβ) plays a dual role in melanoma, mediating tumor suppressive activities at early stages and prooncogenic activities at later stages of tumor progression [1], [2]. At the cell surface, TGF-β binds a complex of transmembrane receptor serine/threonine kinases (types I and II) and induces transphosphorylation and activation of the type I receptor (TβR-I, ALK5) by the type II receptor kinase (TβR-II). The activated type I receptor phosphorylates the downstream effectors Smad2 and Smad3 at C-terminal serines [3], [4], [5]. Smad2 and Smad3 then associate with a common Smad4, and these activated complexes translocate into the nucleus, where they regulate transcription of target genes [6], [7]. The linker region of Smad2 and Smad3, between the MH1 (N-terminal) and MH2 (C-terminal) domains, has been shown to be the target of mitogen-activated protein kinases (MAPK), including ERK, JNK and p38, cyclin-dependent kinases (CDK) and glycogen synthase kinase 3β (GSK3β). Four sites within the linker region have been the focus of several studies: Threonine 220 and Serines 245, 250 and 255 for Smad2; Threonine 179 and Serines 204, 208 and 213 for Smad3 [8], [9], [10], [11], [12], [13], [14], [15], [16], [17], [18], [19], [20]. Although it is now clear that modulation of Smad activity occurs through this linker region, the exact consequences of linker phosphorylation of Smad2 and Smad3 on their transcriptional activity is certainly linked to Smad-interacting partners and the complexity of the promoters.

From studies on epithelial cells, carcinomas, gliomas and melanomas, it appears that Smads, through their linker domain, are at the point of convergence of major cellular signaling pathways, involving ERK, JNK, p38, CDK, GSK3β. GSK3β activity is negatively regulated upon AKT phosphorylation on serine 9 [21]. Therefore, it appeared that a crosstalk between the TGFβ signaling pathway and the AKT/GSK3β arm could take place through the Smad phosphorylation at their linker domain. These results altogether suggested that drugs inhibiting AKT activity could positively regulate GSK3β activity and subsequently increase the Smad linker phosphorylation, and modulate TGFβ signaling.

One drug that can inhibit AKT activity is riluzole, an inhibitor of glutamate release and a FDA approved therapeutic agent for the treatment of amyotrophic lateral sclerosis [22]. Riluzole has shown the ability to inhibit melanoma cell xenograft growth [23], [24] as well as promising results in phase 0 and phase II clinical trials as a single agent in patients with melanoma [25], [26], [27]. The rationale for using riluzole in preclinical studies and melanoma patient trials derived from compelling evidence that the metabotropic glutamate receptor 1 (GRM1), one of the glutamate receptors, was able to induce melanoma in transgenic mice. In addition, the aberrant expression of GRM1 in approximately 65% of melanoma biopsies and cell lines reinforced the hypothesis that GRM1 could become a therapeutic target in melanoma therapy [28].

The present study was designed to determine whether riluzole treatment could result in GSK3β-mediated linker phosphorylation of Smad2 and Smad3, through the inhibition of AKT activity. We present evidence that riluzole inhibits AKT-mediated GSK3β phosphorylation, and increases Smad2 and Smad3 linker phosphorylation by a mechanism involving GSK3β. We also show that riluzole upregulates the expression of two genes associated with the TGFβ signaling pathway, INHBB (coding for Inhibin beta B) and PLAU (coding for the urokinase plasminogen activator).

## Materials and Methods

### Cell Lines

WM793, WM278, and 1205LU were kindly provided by Dr. M. Herlyn (Wistar Institute, Philadelphia, PA, USA [29]). These lines were cultured in MCDB153/L-15 (4/1 ratio) medium containing 2% FBS, 5 µg/ml Insulin and 1.7 mM Calcium Chloride. C8161 melanoma cell line was provided by Dr. Mary Hendrix (Children’s Memorial Research Center, Chicago, IL, USA [30] and was grown in D-MEM (Mediatech, 10-013-CV) containing 10% FBS. UACC930 cells and UACC903 cells were provided by Dr. Jeffrey M. Trent (Translational Genomics Research Center, Phoenix, AZ, USA [31]) and were grown in RPMI1640 (Invitrogen, 11875) containing 10% FBS. The A2058 melanoma cell line was purchased from ATCC (American Type Culture Collection, Manassas, VA 20110, U.S.A) and grown in RPMI1640 containing 10% FBS [25].

### Reagents

Riluzole was purchased from Tocris Biosciences. Lithium Cloride (LiCl) and SB431542 were purchased from Sigma-Aldrich. CHIR-99021 (CT99021) was purchased from Selleck Chemicals (Houston, TX, U.S.A.). Recombinant human TGFβ1 was purchased from R&D systems, Inc (Minneapolis, MN, U.S.A.).

### Treatments

Cells were seeded in 35 mm dishes or 6-well plates (4−5×10^5^/dish or well). The following day, cells were serum-starved for about 16 hours before being incubated with 50 mM Nacl or 50 mM LiCl for 2 and 5 hours. For CT99021 experiment, serum-starved cells were incubated in the presence of DMSO or 2 µM CT99021 for two hours. For riluzole treatment, serum-starved cells were incubated with or without 25 µM riluzole for 9 hours in the absence or presence of either GSK3 inhibitor: LiCl (50 mM) or CT99021 (2 µM). For the TGFβ treatment, serum-starved cells were incubated in the absence or presence of 200 pM TGFβ for 1 or 2 hours.

In the experiments with riluzole, TGFβ and SB431542, serum-starved cells were incubated first with or without 10 µM SB431542 for 1 hour, and then incubated with 25 µM riluzole, 200 pM TGFβ alone or in combination for 9 hours.

### SiRNA GSK3α/β knock-down and Riluzole Treatment

The WM793 melanoma cell line was grown to 80% confluency. 3.10^6^ cells were used for each transfection. Using the Nucleofector™ technology (Amaxa, Lonza), WM793 cells were transfected with 50 nM non targeting control siRNA or GSK3α/β siRNA (Cell Signaling). The cells were then resuspended in warm media and split into two wells of a 6 well tissue culture plate and placed in the 37°C incubator overnight. The following day, the medium was replaced with fresh culture medium for recovery. After overnight serum starvation, cells were incubated with or without 25 µM riluzole for 9 hours before protein extraction.

### *In Vitro* Kinase Assay

Recombinant GST Smad2 and GST Smad3 fusion proteins (3 µg) and 1 µl of GSK3β (New England Biolabs) were incubated at 30°C for 1 hour in a 30 µl reaction containing 20 mM Tris-HCl (pH 7.5), 10 mM MgCl2, 5 mM DTT and 0.5 mM ATP. After 1 hour incubation, SDS sample buffer was added to terminate the kinase reactions. The reaction products were then analyzed by immunoblotting using the pSmad2 (S245/250/255) and pSmad3 (S204) phosphopeptide antibodies.

#### Immunoblotting

Cells were harvested, washed with phosphate-buffered saline, and extracted in the presence of protease and phosphatase inhibitors (Roche) as previously described [10]. Equal amounts of protein were subjected to polyacrylamide gel electrophoresis. After transfer onto nitrocellulose membranes, immunoblots were performed using antibodies against: Both phosphoSmad3 (Thr179) and phosphoSmad2 (Thr220); phosphoSmad3 (Ser204); phosphoSmad3 (Ser208) kindly provided by Dr F. Liu (Center for Advanced Biotechnology and Medicine, Piscataway, NJ, USA) [15]; phosphoSmad2 (Ser245/250/255); Smad2; Smad3. phosphoβ-catenin (Ser33/37/Thr41); β-catenin; GAPDH; phosphoAKT (S473 or T308); AKT; phosphoGSK3 (S9/S21); GSK3 (Purchased from Cell Signaling);

### Construction of GRM1 Over-expressing UACC903 Cell Lines

UACC903 cells were stably transfected with the pcDNA6-GRM1 construct (Yu Wen, Jiadong Li, Seung-Shick Shin, Yong Lin, Byeong-Seon Jeong, Suzie Chen, Karine Cohen-Solal, James Goydos, manuscript submitted) by following the lipofectin (cat. No.1829037 of Invitrogen, Carlsbad, CA) transfection manual provided. Stable clones UACC903-G2 (abbreviated as G2) and UACC903-G4 (abbreviated as G4) over-expressing GRM1 were propagated under selection of 10 ug/ml Blasticidin in RPMI 1640 containing 10% FBS. Control cell line UACC903-V1 (abbreviated as V1) was derived from UACC903 transfected with pcDNA6/V5-HisA empty vector.

### RNA Isolation, TGFß/BMP Signaling Pathway PCR Array and qPCR Validation

Total RNA was isolated using Trizol (Invitrogen, Carlsbad, CA) and Direct-zol RNA miniprep kit (Zymo Research, Irvine, CA) following manufacturer’s instruction. One microgram of total RNA was used for cDNA synthesis using SuperScript II cDNA synthesis kit (Invitrogen, Carlsbad, CA) for standart qPCR and RT^2^ first strand kit (SABiosciences, Qiagen, Valencia, CA) for the TFGβ**/**BMP signaling pathway PCR arrays. For the TFGβ**/**BMP signaling pathway PCR array, the cDNAs were added to the RT^2^ qPCR master mix, and the mixture was aliquoted across the PCR array, according to the manufacturer recommendations. The qPCR was performed in One Step Plus qPCR instrument (Applied Biosystems Inc, Carlsbad, CA). All primers for SYBR qRT-PCR were purchased from Qiagen (Valencia, CA). Changes in gene expression were calculated using the delta delta Ct method. All experiments were independently replicated 3 times.

## Results

### GSK3 is Involved in Smad Phosphorylation at the Linker Domain

**Figure 1:**
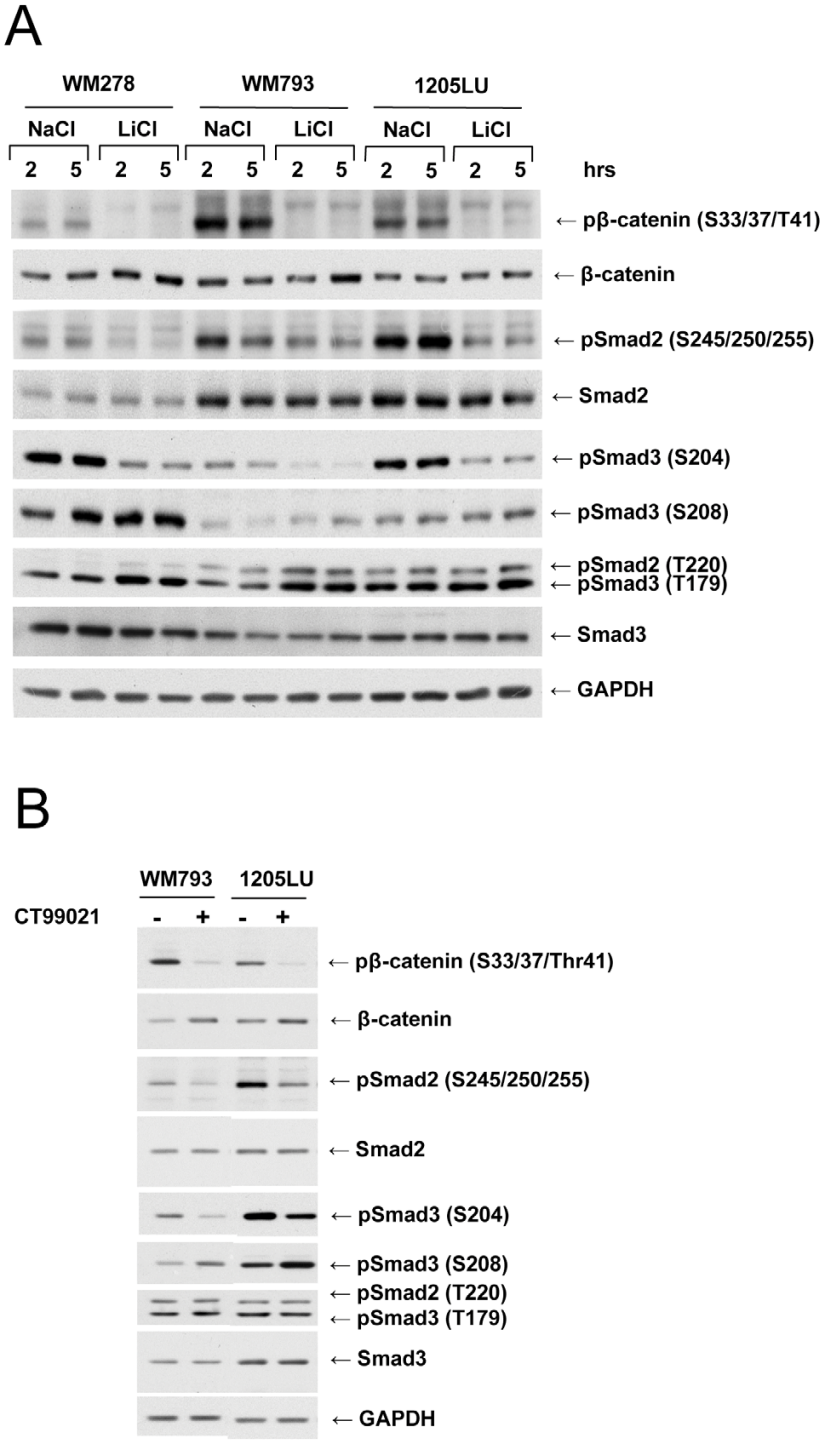
Smad linker phosphorylation involves GSK3 in human melanoma lines. A. Inhibition of Smad2 and Smad3 linker phosphorylation in the presence of LiCl. 24 hours post seeding, WM278, WM793 and 1205LU human melanoma cell lines were serum-starved for about 16 hours, before the addition of 50 mM LiCl for 2 and 5 hours. Cells left with 50 mM NaCl for 2 or 5 hours were used as controls. Whole cell extracts were then prepared. Immunoblots were performed with antibodies against: Phosphorylated β-catenin (pβ-catenin); total β-catenin; Both phosphoSmad3 (Thr179) and phosphoSmad2 (Thr220); phosphoSmad2 (Ser245/250/255); phosphoSmad3 (Ser204); phosphoSmad3 (Ser208); Smad2 and Smad3; GAPDH. **B.** Inhibition of Smad2 and Smad3 linker phosphorylation in the presence of the specific GSK3 specific inhibitor, CT99021. 24 hours post seeding, WM793 and 1205LU cells were serum-starved for about 16 hours, and incubated in the absence (−) or presence (+) of 2 µM of CT99021 for two hours. Immunoblots were performed as in A. p: Phosphorylated. S: Serine; T: Threonine.

We previously demonstrated that the two pan-CDK/GSK3 inhibitors, flavopiridol [32], [33], [34] and R547 [33], [35], [36] could inhibit the constitutive linker phosphorylation of Smad2 and Smad3 in melanoma cell lines [10]. In order to determine whether GSK3 was involved in Smad2 and Smad3 linker phosphorylation, we used two different types of GSK3 inhibitors: Lithium Chloride and the GSK3 specific inhibitor CT99021 [37]. The effect of LiCl treatment was assessed first on β-catenin phosphorylation at Ser33/37/Thr41, which are GSK3 sites [38], [39]. As shown in Figure 1A, treatment of human melanoma cell lines with LiCl resulted in inhibition of β-catenin phosphorylation at these sites. We also observed a slight increase in β-catenin as a result of its subsequent stabilization. Phosphorylation of Smad2 at the cluster of serines (245/250/255) was inhibited in the presence of LiCl at the two time points, 2 and 5 hours, and for the three melanoma cell lines. In addition, phosphorylation of Smad3 at serine 204 was also inhibited in the presence of LiCl similarly for 2 and 5 hours treatment. Therefore, GSK3 likely plays a role in Smad linker phosphorylation, at the cluster of serines in Smad2 and at serine 204 in Smad3. In contrast, there was a slight increase of phosphorylation at serine 208 and threonine 179 in Smad3 and threonine 220 in Smad2, in the presence of LiCl, suggesting that GSK3 normally inhibits the function of one or more kinase(s) involved in the phosphorylation at these sites. To confirm the involvement of GSK3, we used a specific inhibitor of GSK3, CT99021 [40]. As shown in Figure 1B, phoshorylation of β-catenin is decreased and total β-catenin level is slightly increased as a result of CT99021 2-hour treatment. Smad2 phosphorylation at the cluster of serines (245/250/255) and Smad3 phosphorylation at serine 204 are decreased after CT99021 treatment, while phosphoSmad3 (S208) levels are increased. Phosphorylation at threonines 179 (Smad3) and 220 (Smad2) are not affected by CT99021 treatment. These results strongly suggest that GSK3 is implicated in the Smad2 and Smad3 linker phosphorylation, at the cluster of serines and at serine 204 respectively.

### Riluzole Decrease AKT Phosphorylation and AKT-mediated GSK3 Phosphorylation

**Figure 2:**
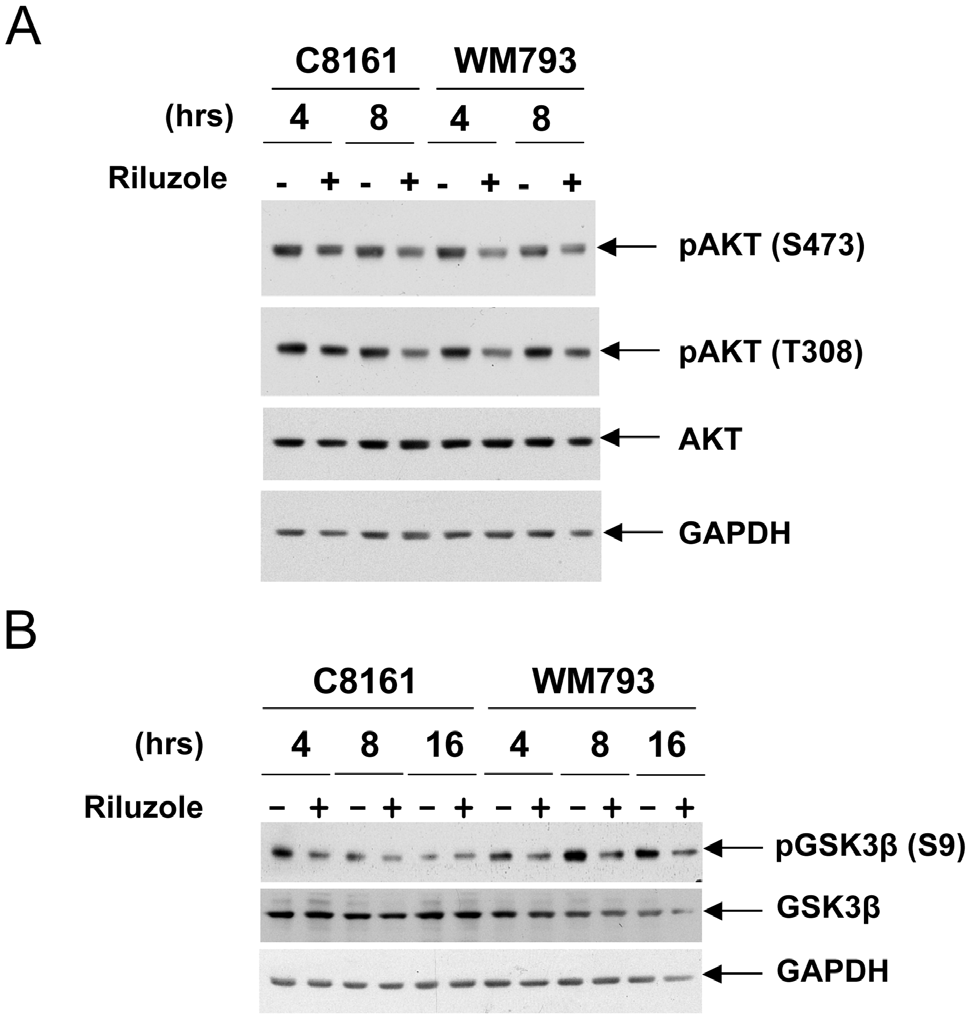
Riluzole decreases AKT phosphorylation and activity. A. Riluzole decreases AKT phosphorylation. Serum-starved melanoma cells were incubated in the absence (−) or presence (+) of 25 µM of riluzole for 4 and 8 hours. Immunoblots were performed using antibodies against AKT phosphorylated at serine 473 (pAKT (S473)) or at threonine 308 (pAKT (T308)). Anti-total AKT and GAPDH antibodies were used for normalization. **B.** AKT-mediated GSK3 phosphorylation is decreased after riluzole treatment. Serum-starved melanoma cells were incubated in the absence (−) or presence (+) of 25 µM of riluzole for 4, 8 and 16 hours. Immunoblots were performed using antibodies against GSK3β phosphorylated at serine 9, an AKT site; total GSK3β; GAPDH.

We previously reported that riluzole inhibited phosphorylation of AKT, suggesting that this agent could negatively affect AKT activity [25]. As shown in Figure 2A, riluzole decreases AKT phosphorylation on serine 473 and threonine 308. AKT phosphorylates GSK3 on serine 9 for GSK3β or 21 for GSK3α, thereby inactivating GSK3 [21]. Therefore, we hypothesized that by inhibiting AKT activity, riluzole could decrease the phosphorylation of GSK3 at the AKT site. In order to determine whether GSK3 phosphorylation at the AKT site was decreased in the presence of riluzole, melanoma cells were incubated in the absence or presence of this agent for 4, 8 and 16 hours and GSK3β phosphorylation at the AKT site was analyzed. As shown in Figure 2B, treatment of melanoma cells with riluzole led to a decrease in AKT-mediated GSK3β phosphorylation on serine 9. These results suggest that riluzole could positively regulate GSK3β activity. Since we showed that GSK3β is involved in phosphorylating the Smad linker domain, we investigated whether riluzole was able to induce an increase in Smad linker phosphorylation.

### Riluzole Increases Smad2 Linker Phosphorylation at the Cluster of Serines and Smad3 Linker Phosphorylation at Serine 204 through GSK3

**Figure 3:**
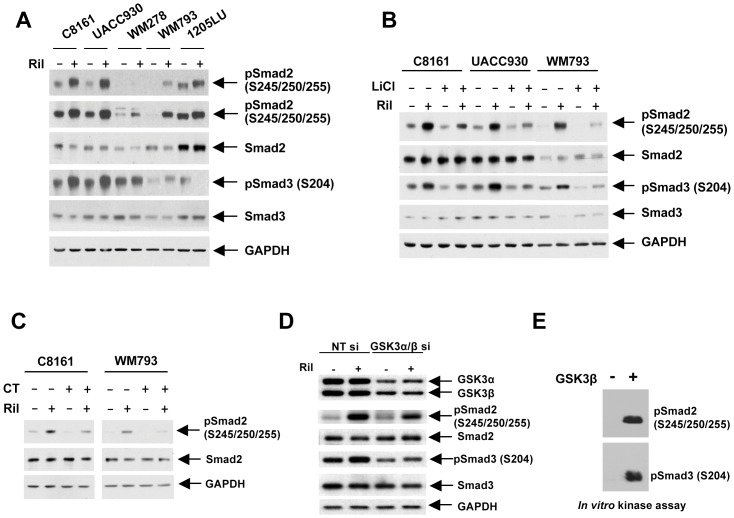
Riluzole increases Smad2 linker phosphorylation at the cluster of serines and Smad3 linker phosphorylation at serine 204 through GSK3. **A.** Riluzole increases Smad2 and Smad3 linker phosphorylation. 24 hours post seeding, C8161, UACC930, WM278, WM793 and 1205LU human melanoma cell lines were serum-starved for about 16 hours, and incubated in the absence (−) or presence (+) of 25 µM of riluzole for 9 hours. Immunoblots were performed using phosphoSmad2 (Ser245/250/255); phosphoSmad3 (Ser204); Smad2 and Smad3; GAPDH. Two exposures are presented for phosphoSmad2 (Ser245/250/255) to see the lower signals for WM278. **B.** LiCl treatment counteracts riluzole-induced Smad linker phosphorylation. After serum starvation, cells were incubated in the absence (−) or presence (+) of 25 µM riluzole either in the presence of NaCl (−) or LiCl (+) for 9 hours. Immunoblots were performed as in A. **C.** Treatment with the specific GSK3 inhibitor, CT99021, counteracts riluzole-induced Smad linker phosphorylation. After serum starvation, cells were incubated in the absence (−) or presence (+) of 25 µM riluzole either in the absence (−) or presence (+) of CT99021 (CT) for 9 hours. Immunoblots were performed as in A. **D.** GSK3α and GSK3β knock-down inhibits riluzole-induced Smad linker phosphorylation. WM793 melanoma cells were transfected with non targeting control siRNA (NT si) or GSK3α/β siRNA (GSK3α/β si), serum-starved and incubated with or without riluzole for 9 hours before protein extraction. Immunoblots were done as in A. **E.** GSK3β can phosphorylate the cluster of serines 245/250/255 in Smad2 and serine 204 in Smad3 *in vitro*. Recombinant GSK3β was used to phosphorylate GST-Smad2 and GST-Smad3 in a non radioactive reaction. The reaction products were analyzed by immunoblotting as in A. Ril: Riluzole.

In order to determine whether riluzole could increase Smad linker phosphorylation, melanoma cell lines were incubated in the presence of this agent for 9 hours. As shown in Figure 3A, riluzole-treated cells had increased linker phosphorylation of Smad2 at serines 245/250/255 in the five melanoma cell lines tested and of Smad3 at serine 204 in all but the 1205LU cell line. As previously shown (Figure 1), constitutive phosphorylation of serines 245/250/255 in Smad2 and serine 204 in Smad3 involves GSK3 activity. To directly demonstrate that GSK3 mediated the Smad linker phosphorylation induced by riluzole, melanoma cell lines were treated with riluzole, in the absence or presence of pharmacological inhibitors of GSK3, LiCl and CT99021. As shown in Figures 3B (LiCl) and 3C (CT99021), GSK3 inhibition led to reduction of basal and riluzole-induced phosphorylation of Smad2 and Smad3 linker phosphorylation. In addition, siRNA knock-down of GSK3α and GSK3β inhibited the riluzole-induced phosphorylation of Smad2 (S245/250/255) and Smad3 (S204) (Figure 3D). Finally, the same sites were robustly phosphorylated by GSK3β in an *in vitro* kinase assay (Figure 3E). These results strongly suggest that riluzole, by successively inhibiting AKT and activating GSK3 activities, increases Smad linker phosphorylation.

### Riluzole does not Activate the TGFβ/TGFβ Receptor Complexes to Induce Smad Linker Phosphorylation

**Figure 4:**
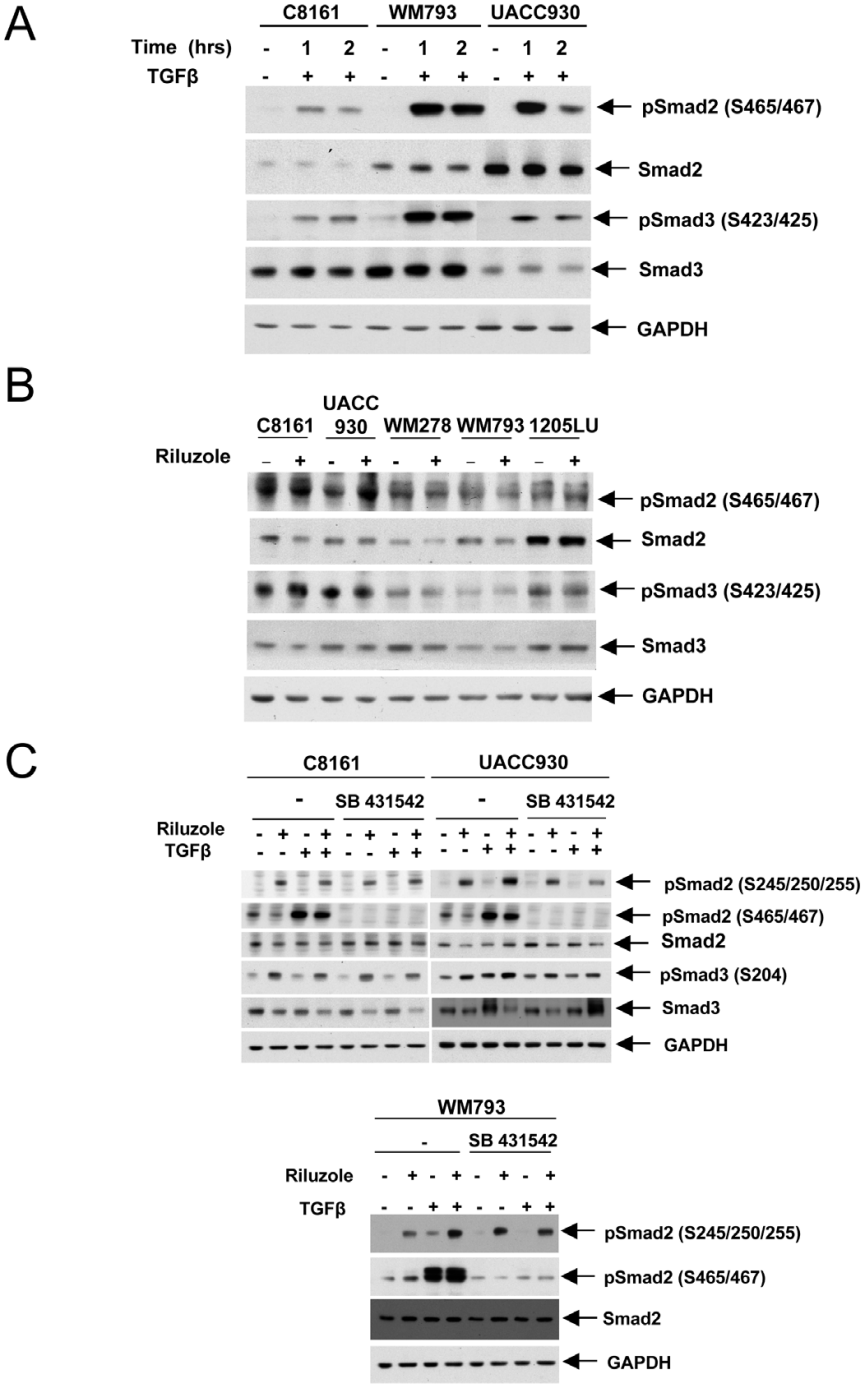
Riluzole effect on Smad linker phosphorylation does not involve the activation of the TGFβ canonical signaling pathway. **A.** Increased C-terminal phosphorylation in the presence of TGFβ. Serum-starved cells were incubated in the absence (−) or presence (+) of TGFβ for 1 and 2 hours. Immunoblots with antibodies against the C-terminal phosphorylated form of Smad2 (pSmad2(S465/467)) or Smad3 (pSmad3(S423/425)); total Smad2 and Smad3; GAPDH. **B.** Riluzole does not affect the C-terminal phosphorylation of Smads. After serum starvation, cells were incubated in the absence (−) or presence (+) of riluzole for 9 hours. Immunoblots were done as in A. **C.** TβRI inhibition by SB431542 does not decrease riluzole-induced Smad linker phosphorylation. Serum-starved C8161, UACC930 and WM793 cells were incubated in the absence (−) or presence (+) of SB431542 for 1 hour, before incubation with riluzole and TGFβ, alone or in combination for 9 hours. Immunoblots were performed using antibodies against the linker phosphorylated form of Smad2 (pSmad2 (S245/250/255)); the C-terminal phosphorylated form of Smad2 (pSmad2(S465/467)); total Smad2; the linker phosphorylated form of Smad3 (pSmad3 (S204); total Smad3 and GAPDH.

It was reported that TGFβ induced Smad3 linker phosphorylation at serine 204, 208 and threonine 179, and that GSK3 was responsible for the TGFβ-inducible serine 204 phosphorylation, by a mechanism yet to be determined. The TGFβ-inducible serine 204 phosphorylation required prior activation of the canonical TGFβ signaling pathway leading to Smad3 C-terminal phosphorylation [17], [20]. We therefore asked whether, in addition to a decrease in AKT-mediated GSK3 phosphorylation and subsequent GSK3 activation, riluzole could increase Smad linker phosphorylation by an additional mechanism. Indeed, riluzole treatment could also result in the activation of the TGFβ canonical signaling pathway, subsequent GSK3 activation and Smad linker phosphorylation. We first verified that the human melanoma lines were responding to TGFβ, as measured by an increase in the C-terminal phosphorylation of Smad2 and Smad3 (S465/467 and S423/425 respectively) upon TGFβ treatment (Figure 4A). In order to determine whether riluzole could activate the TGFβ canonical signaling pathway, we analyzed Smad2 and Smad3 C-terminal phosphorylation in the absence and presence of riluzole. As shown in Figure 4B, we did not observe any increase in Smad C-terminal phosphorylation in the presence of riluzole, suggesting that riluzole does not activate the canonical TGFβ signaling pathway. To directly address the role of the TGFβ signaling pathway in the GSK3-dependent effect of riluzole on Smad linker phosphoryation, we used an inhibitor of the TGFβ superfamily type I activin receptor-like kinase (ALK) receptors, SB431542. SB431542 should inhibit the activation of TGFβ receptor I resulting in an inhibition of the canonical TGFβ signaling pathway and a decrease in Smad2 and Smad3 C-terminal phosphorylation. As shown in figure 4C, we showed that the TGFβ-induced C-terminal phosphorylation of Smad2 (pSmad2 (S465/467)) was abolished in the presence of SB431542 in C8161, UACC930 and WM793 melanoma cell lines, as expected. Interestingly, the TGFβ-induced Smad2 linker phosphorylation (pSmad2 (S245/250/255), visible in WM793 cells, was also inhibited in the presence of SB431542, suggesting that the TGFβ-induced Smad2 linker phosphorylation required prior C-terminal phosphorylation of Smad2 in these cells. These results are in accordance with TGFβ-induced Smad3 linker phosphorylation requiring prior Smad3 C-terminal phosphorylation [17], [20]. In contrast, riluzole-induced Smad2 linker phosphorylation was not inhibited in the presence of SB431542, indicating that riluzole effect does not require activation of the TGFβ canonical signaling pathway to increase Smad linker phosphorylation. We obtained the same results in C8161 and UACC930 human melanoma cell lines; SB431542 did not inhibit riluzole-induced Smad2 and Smad3 linker phosphorylation in these lines. These results suggest that the only mechanism by which riluzole induces Smad linker phosphorylation is through inhibition of AKT-mediated GSK3 phosphorylation and subsequent activation of GSK3.

### The Extent of Riluzole-induced Smad Linker Phosphorylation is Independent of GRM1 Expression in Melanoma Cells

**Figure 5:**
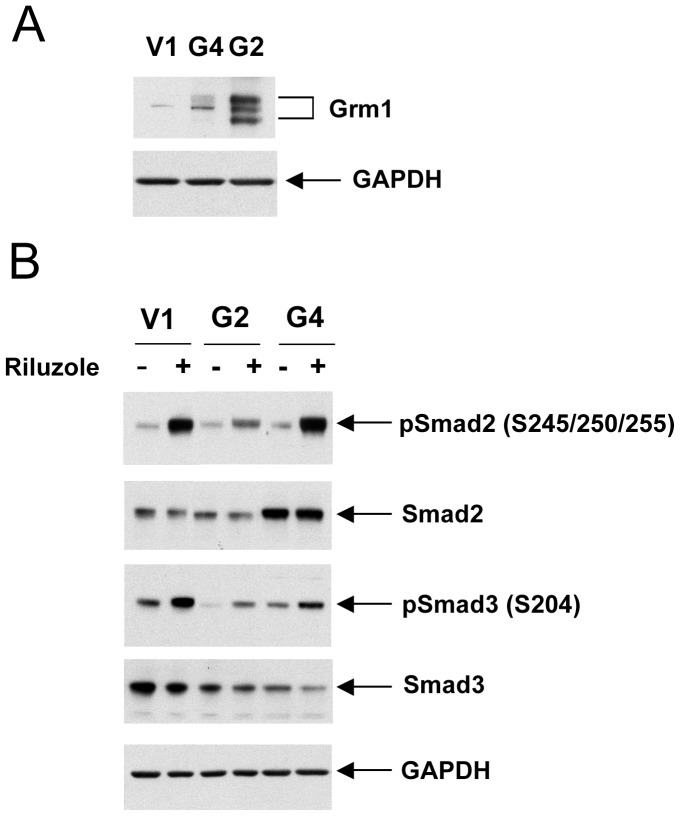
Riluzole-induced Smad linker phosphorylation is independent of GRM1 expression level in melanoma cells. A. Immunoblot with lysates from UACC903-V1 (V1) cells and two clones overexpressing GRM1, called UACC903-G2 and UACC903-G4 (abbreviated as G2 and G4 respectively). **B.** Riluzole-induced Smad2 and Smad3 linker phosphorylation in V1, G2 and G4 cells.

We previously mentioned that preclinical studies *in vitro* and animal models pointed to the metabotropic glutamate receptor 1 (GRM1) as a key player in melanoma development [24], [28], [41]. The observation that melanoma cell lines released high levels of glutamate, resulting in a glutamate-based autocrine activation of GRM1 [24], prompted our group to consider riluzole as a potential therapeutic agent acting through the inhibition of glutamate release from melanoma cells [23], [24], [25], [27], [42]. However, riluzole’s effect might not be mediated through inactivation of the glutamate/GRM1-based autocrine loop. Other glutamate-based autocrine loops potentially exist in melanoma cells. Melanoma cells express and exhibit dysregulation of other glutamate receptors, including other metabotropic glutamate receptors [43], [44], [45] and the ionotropic glutamate receptors [46]. In order to determine whether riluzole’s effect on Smad linker phosphorylation was mediated by inactivation of the GRM1 signaling pathway, we used UACC903 cells that express a low level of GRM1 and stably transfected these cells with a GRM1 expression vector (Yu Wen, Jiadong Li, Seung-Shick Shin, Yong Lin, Byeong-Seon Jeong, Suzie Chen, Karine Cohen-Solal, James Goydos, manuscript submitted). Two clones called UACC903-G2 and UACC903-G4 (abbreviated as G2 and G4 respectively), had significantly higher GRM1 expression levels than a vector control cell line UACC903-V1 (V1) (Figure 5A). In V1, G2 and G4 cell lines, riluzole induced Smad2 and Smad3 linker phosphorylation independently of the GRM1 expression level, indicating that the riluzole effect was not directly related to the level of GRM1 expression in these cell lines. (Figure 5B). In addition, in the UACC930 melanoma cell line, where the expression of GRM1 protein is not detectable [24], the induction of Smad2 and Smad3 linker phosphorylation in the presence of riluzole is comparable to that of the other melanoma cell lines that express GRM1 (Figures 3A and 3B). These results suggest that riluzole-induced Smad linker phosphorylation in melanoma cells is not directly related to GRM1 expression.

### Riluzole Upregulates the Expression of INHBB and PLAU

**Figure 6:**
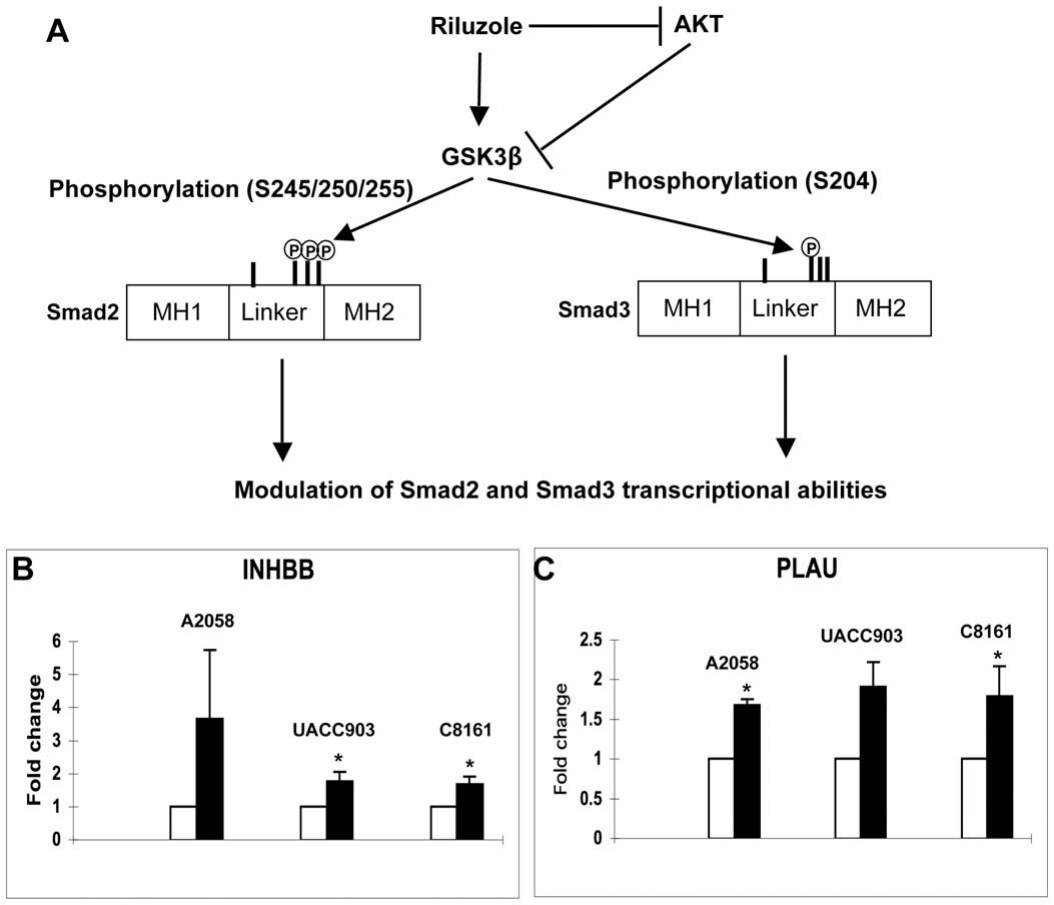
Riluzole upregulates the expression of genes associated with the TGFβ signaling pathway. **A.** Model of riluzole effect on Smad linker phosphorylation. Riluzole inhibits the phosphorylation of AKT at S473 and T308. As a result of AKT inactivation, GSK3 phosphorylation at the AKT site is decreased, resulting in GSK3 activation. GSK3 phosphorylates Smad2 at the cluster of serines 245/250/255 and Smad3 at serine 204. The consequences of these phosphorylation events on Smad2 and Smad3 activities will likely depend on the promoters of the TGFβ target genes. **B and C.** Riluzole treatment increases the expression of the INHBB and PLAU genes respectively in the three independent melanoma cell lines A2058, UACC903 and C8161. mRNA from these three melanoma cell lines untreated or treated with riluzole for 24 hours were analyzed by real-time PCR for the expression of the INHBB and PLAU genes. White bars: untreated cells. Dark bars: Riluzole-treated cells. *, P<0.05, compared with untreated cells (t test).

The positive effect of riluzole on Smad2 and Smad3 linker phosphorylation (Figure 6A) prompted us to investigate the potential action of Riluzole on the expression of TGFβ target genes. We used human TGFß/BMP Signaling Pathway RT^2^ Profiler™ PCR Arrays to identify known TGFβ target genes whose expression could be modulated by riluzole. For this purpose, RNAs were isolated from WM793 melanoma cells incubated in the absence or presence of riluzole. cDNAs converted from these RNAs were processed for real-time PCR using one PCR array for the untreated cells-derived cDNA and one array for the riluzole-treated cells-derived cDNA. By comparing the two, we identified four genes whose expression was increased or decreased in the presence of riluzole as compared with untreated WM793 melanoma cells (Table 1). By qPCR, we validated the upregulation of two genes, INHBB and PLAU, in three additional melanoma cell lines (Figure 6B and C). The INHBB gene codes for the inhibin, beta B chain. Two beta B subunits form a homodimer called activin B. Activin is a member of the TGFβ superfamily [47]. PLAU codes for the urokinase plasminogen activator (uPA), belonging to the urokinase plasminogen activating system (uPAS). The TGFβ signaling positively regulates uPA expression, secretion and stability through the Smad-dependent pathway [48], [49]. Therefore riluzole positively regulates the expression of genes associated with the TGFβ signaling pathway.

**Table 1:** Genes whose expression was regulated by riluzole in the WM793 cell line using the Human TGFβ/BMP Signaling Pathway RT^2^ Profiler™ PCR Array.

Gene symbol	Fold regulation
BMP3	−2.46
GDF5	3.35
INHBB	2.97
PLAU	3.69

The fold increase or decrease (comparing to control) was above 2 for these four genes. BMP3: bone morphogenetic protein 3; GDF5: growth differentiation factor 5; INHBB: inhibin, beta B; PLAU: plasminogen activator, urokinase.

## Discussion

In the present study, we described a cross-talk between three pathways involved in melanoma biology, the TGFβ signaling pathway [1], [2], the AKT/GSK3 pathway [50] and the glutamate signaling [23], [24], [25], [27], [42], [51]. We demonstrated that the glutamate release inhibitor riluzole inhibited AKT phosphorylation at crucial sites for its activity, and consequently inhibited GSK3β phosphorylation at an AKT site, thereby activating GSK3β. We also provided evidence for the involvement of GSK3 in basal and riluzole-induced linker phosphorylation of Smad2 and Smad3. Indeed, treatment with two types of GSK3 inhibitors and the GSK3α/β knock-down counteracted basal and riluzole-induced Smad2 and Smad3 linker phosphorylation. In addition, *in vitro* kinase assays confirmed that GSK3β could phosphorylate Smad2 and Smad3 at the cluster of serines 245/250/255 and serine 204 respectively.

Interestingly, we also demonstrate riluzole induced Smad linker phosphorylation in the GRM1 negative UACC930 melanoma cell line [24] (this line does not express GRM1 because of a truncation mutation [S. Chen, personal communication]). In this melanoma cell line, like in the others, GSK3 mediated the riluzole-induced Smad linker phosphorylation. However, we previously described that riluzole treatment did not inhibit AKT phosphorylation in UACC930 cells [25]. Therefore, in this line, the activation of GSK3 in the presence of riluzole would involve a different mechanism, such as Wnt signaling inactivation. These results are in apparent contradiction with a study describing riluzole as an enhancer of Wnt/β-catenin signaling and GRM1 as the likely target of riluzole-mediated enhancement of Wnt/β-catenin in the human melanoma cell line A375 and the mouse melanoma cell line B16 [51]. This group also found that riluzole did not induce a decrease in ERK phosphorylation in the A375 melanoma cell line, in contrast to the decrease in phosphorylated ERK in all human melanoma cell lines positive for GRM1 [25], [27]. From these results, it is likely that genetic and epigenetic context-dependent responses can be expected when treating melanoma cell lines with riluzole, as already suggested by the mixed responses to riluzole and the failure of some patients to respond to riluzole in clinical trials, independently of GRM1 expression [26], [27].

TGFβ-induced Smad linker phosphorylation has been described in a wide variety of cellular systems, including melanoma cells. The different kinases involved in each of these studies include JNK, CDKs, GSK3, depending on the phosphorylation site and the cellular context [8], [9], [11], [17], [18], [19], [20], [52]. We have shown that riluzole-induced Smad linker phosphorylation is mechanistically different from the TGFβ-induced Smad linker phosphorylation. First, riluzole does not induce C-terminal Smad phosphorylation, suggesting that the TGFβ/receptor complexes are not engaged upon riluzole treatment. In contrast, the initial step after TGFβ activation is the C-terminal phosphorylation of Smad2 and Smad3, and this step is required for the TGFβ-induced Smad3 phosphorylation [20]. Second, riluzole does not affect the expression of TGFβ1, TGFβ2 or TGFβ3 as shown by real time RT-PCR (data not shown). Therefore, this does not support the possible hypothesis that riluzole increases Smad linker phosphorylation by inducing TGFβ production. Finally, in contrast to TGFβ-induced Smad linker phosphorylation, the TβRI inhibitor, SB431542, did not inhibit the riluzole-induced Smad linker phosphorylation.

The effect of riluzole on the linker phosphorylation of Smad2 and Smad3, downstream effectors of TGFβ, will likely modulate TGFβ signaling and the expression of TGFβ target genes. Our previous report suggested that Smad3 linker phosphorylation might contribute to the resistance to TGFβ-mediated cell growth inhibition in melanoma, by inhibiting the expression of p15 and p21. However, Smad3 linker phosphorylation did not inhibit the expression of PAI-1, involved in TGFβ pro-oncogenic effects. Therefore, Smad3 activity would be inhibited on promoters involved in cell growth inhibition, such as p15 and p21, but fully competent for regulating some of the genes involved in TGFβ prooncogenic effects [10]. This model is in accordance with the well-documented fact that not all Smad transcriptional activities have been disrupted in melanoma cells [1]. It is now clear that Smad transcriptional activities are modulated by phosphorylation at their linker domain, but the nature of this modulation will likely depend on the promoter of each TGFβ target gene, of the other transcription factors (repressors, activators), binding this promoter and the consequences of these phosphorylation events on the interaction between linker phosphorylated Smad and these other transcription factors. In addition, the identity of the sites phosphorylated in Smad2 and Smad3 (Threonine 220, Serines 245,250 and 255 for Smad2; Thr179 and Serines 204, 208 and 213 for Smad3) will play a role in the modulation of the TGFβ target genes. We have shown that riluzole induces the phosphorylation of the cluster of serines (245/250/255) in Smad2 and serine 204 in Smad3 via GSK3, in the majority of the melanoma cell lines analyzed. The exact consequences of these phosphorylation events on Smad2 and Smad3 transcriptional activities will be promoter dependent, as mentioned previously.

We initiated the characterization of genes associated with the TGFβ signaling pathway and whose expression was modulated by riluzole. Our goal was to define possible mediators of riluzole action downstream of the TGFβ signaling pathway. Since TGFβ exerts pro-oncogenic activities at late stages of melanoma development, it is important to determine whether riluzole can have a negative effect on the expression of genes involved in TGFβ pro-oncogenic activities. This could explain the inhibition of melanoma cell growth in mice and the involution of some of the tumors in patients treated with riluzole. Alternatively, a positive regulation of genes involved in TGFβ pro-oncogenic activities by riluzole could explain the mixed responses to riluzole and the failure of some patients to respond to riluzole in clinical trials [26], [27]. We characterized two genes whose expression was upregulated by riluzole. The first one codes for inhibin beta B. Two inhibin beta B subunits form a homodimer called activin B, which is a member of the TGFβ superfamily [47]. The fact that riluzole upregulates the expression of activin B could potentially have a negative impact on riluzole response since activin is suggested to play an active role in several carcinomas and glioma migration, invasion and progression [53], [54], [55], [56], [57]. In addition, one study suggested that melanoma cells might be resistant to the growth inhibitory and pro-apoptotic effects of activin [58]. Resembling the dual function of TGFβ in melanoma, melanoma cells would be resistant to activin-mediated tumor suppression, but would utilize activin to promote their migration and metastasis. The second gene whose expression was upregulated by riluzole was PLAU coding for the urokinase plasminogen activator, described as a TGFβ target gene [48], [49]. It was shown that tumor growth was retarded in uPA-deficient mice [59], [60]. uPA belongs to the uPAS system, which plays multiple roles in the neoplastic evolution, including angiogenesis, tumor cell proliferation, adhesion, migration, intravasation and growth at the metastatic site [61]. As suggested earlier regarding the upregulation of activin expression by riluzole, riluzole positive regulation of PLAU might contribute to the mixed responses to riluzole and the failure of some patients to respond to riluzole in clinical trials [26], [27].

At this point of the study, we cannot directly link the riluzole-induced linker phosphorylation of Smad2 and Smad3 to the upregulation of INHBB and PLAU expression. This would require an extensive analysis using stable transfectants expressing wild-type and Smad mutant forms unable to be phosphorylated in the specific GSK3 sites. However, our study revealed an important cross-talk between three melanoma signaling pathways, the glutamate signaling, the PI3K/AKT pathway and the TGFβ signaling pathway. We then focused on the TGFβ signaling pathway as a possible mediator of riluzole actions. We then identified two genes whose upregulation by riluzole might be detrimental to a more complete response to this agent in clinical trials. The identification of cross-talks such as those described in this study could be instrumental in predicting responses to riluzole-based therapy.
